# New Editor-in-Chief for *Clinical Neuroradiology*

**DOI:** 10.1007/s00062-023-01278-y

**Published:** 2023-03-14

**Authors:** Martin Bendszus

**Affiliations:** Abteilung für Neuroradiologie, Universitätsklinikum Heidelberg, Im Neuenheimer Feld 400, 69120 Heidelberg, Germany

As of 31 December 2022 Prof. Dr. László Solymosi has resigned as the Editor-in-Chief of *Clinical Neuroradiology*. It is a great honor for me to take over this responsibility with the beginning of 2023. First of all, my thanks and respect go to László Solymosi under whom the journal *Clinical Neuroradiology* has evolved from a German language journal with little visibility to an internationally renowned journal in neuroradiology. I had the honor to contribute to this development as a Co-editor from the very beginning of his responsibility as Editor-in-Chief. There are no good reasons to substantially change the overall scope and strategy of the journal. The main focus will remain and be sharpened in the field of interventional neuroradiology, diagnostic neuroradiology, and new evolutions, e.g. artificial intelligence and new diagnostic and interventional developments.

The attractiveness of a scientific journal fundamentally relies on the quality of the published papers. Therefore, my sincere request as new Editor-in-Chief is to submit high-quality scientific work to *Clinical Neuroradiology*. I guarantee a fast and transparent review process and rapid online publication of accepted papers. Likewise, this is substantially dependent on a rapid review by the respective reviewers, who I would explicitly like to thank for their past and future commitment to the journal.

I am very much looking forward to my work as the Editor-in-Chief with the goal to further establish *Clinical Neuroradiology* as a leading journal in diagnostic and interventional neuroradiology.

